# Qualitative study of barriers and facilitators to HIV detection and treatment among women who inject drugs during the war against Ukraine

**DOI:** 10.1186/s12981-023-00578-0

**Published:** 2023-11-13

**Authors:** Olena Karagodina, Oksana Kovtun, Myroslava Filippovych, Oleksandr Neduzhko

**Affiliations:** 1 European Institute of Public Health Policy, LLC, Kyiv, Ukraine; 2 Alliance for Public Health, ICF, Kyiv, Ukraine

## Abstract

**Background:**

The Russian Federation’s invasion in Ukraine has resulted social hardship, millions of internally displaced persons, the destruction of medical infrastructure, and limited access to HIV services. There is no available information regarding the impact of the war on the HIV treatment cascade among women who inject drugs (WWID) in Ukraine. In this study, we examine the barriers and facilitators of HIV detection, initiation of treatment, and adherence to antiretroviral therapy (ART) among WWID.

**Methods:**

During the in-depth interviews, participants were queried about their needs for HIV testing, treatment and related services, as well as barriers to HIV testing, initiation and retention on ART, including organizational barriers and changes in existing preventive and treatment programs. Thematic content analysis was used to employed to derive the results.

**Results:**

From August to September 2022, we conducted in-depth interviews among 38 WWID in Kryvyi Rih, Kyiv, and in the Ivano-Frankivsk and Odesa regions of Ukraine. The most persistent personal facilitator for HIV detection, ART initiation, and retention in services was a combination of several factors, including strong ties with relatives and a sense of responsibility for loved ones, support from the family, willingness to cooperate with specialists, a higher level of education, and a relatively prosperous financial situation. Barriers such as war-related stress and disruptions to healthcare facilities are directly linked to the ongoing war. The influence of other barriers (fear of discovering the presence of the disease, potential social restrictions, and drug use) was universal and only indirectly related to the state of war. The majority of WWID provided positive assessments of the quality of work and the availability of preventive HIV services.

**Conclusion:**

The ongoing war against Ukraine continues to have a detrimental impact on all aspects of the population’s life, particularly affecting WWID. Providers of HIV services must make every effort to sustain and optimize these services, taking into account the evolving context and new requirements. The changing life situation and shifting priorities of WWID necessitate a dynamic and comprehensive assessment of existing challenges.

## Introduction

The full-scale invasion of Ukraine by the Russian Federation began on February 24, 2022, resulting in death, destruction, and widespread human suffering. In 2023, it is estimated that at least 17.6 million people will be require humanitarian assistance in 2023, including 6.3 million of internally displaced persons (IDPs) and 4.4 million who have returned to their homes [1]. Among the 6.3 million IDPs, a majority (59%) are women [1]. These IDPs often lack permanent housing, face unemployment, and have limited access to social support [2]. The destruction of medical facilities, treatment delays or shortages, displacement of people, and limited access to water and sanitation may contribute to an increased incidence of infectious diseases in Ukraine [3].

According to the estimates, there are about 319,500 (172,000–590,500) people who inject drugs (PWID) in Ukraine, of whom women make up about 23.2% (20.8–23.6) [4]. The estimated number of people living with HIV (PLHIV) was 245,000 people in Ukraine (215,000–281,000) as of the end of 2021 [5]. At the same time, only 150,005 HIV-positive citizens of Ukraine and 262 foreigners were enrolled in medical care in health care facilities (HCFs) – about 47% of PLHIV [5]. According to estimated data, about 19.1% (16.1–22.2%) of the total number of PWID are living with HIV [4]. With the support of the ICF “Alliance for Public Health” in cooperation with non-governmental organizations (NGOs), a number of programs are being implemented among PWID in Ukraine to identify PLHIV, timely initiate antiretroviral therapy (ART) and to improve adherence to treatment. A free HIV prevention package for PWID includes condoms, lubricants, counseling, and testing for HIV, syphilis, hepatitis C, as well as early tuberculosis screening [6, 7]. Primary medical care and HIV care are also provided free of charge in Ukraine as part of the guaranteed medical package [8, 9]. The HIV prevention and treatment programs have yielded tangible results. The findings from the biobehavioral study (BBS) conducted among PWID in 2020 demonstrate a decrease in HIV prevalence among PWID from 22.6% to 2017 to 20.3%, and a decrease in incidence from 2.4–1.06% [10]. Over the course of three years, the cascade of HIV treatment among PWID also improved at all stages. In 2017, the indicators were 58-91-73-74 (aware of their HIV-positive status – enrolled in care – receive ART – have reached an undetectable level of viral load). By 2020, these indicators had improved to 64-94-92-82 [7, 10].

Women who inject drugs (WWID) are at a heightened risk of HIV infection [11]. The results of the 2020 BBS among PWID reveal variations in HIV prevalence based on gender and age [10]. Notably, the prevalence of HIV infection among WWID is 29.6%, whereas it is 18.1% among men. Furthermore, the cascade of treatment for HIV-positive women demonstrates a sequence of 67-96-91-82, whereas for men, it is 63-94-92-80. However, it’s important to note that these statistics may not fully represent the HIV situation among WWID, as their proportion in the sample was 19%, and they are often considered hard-to-reach for research projects. The prevalence of HIV increases with age, reaching 32.6% for PWID over 45 years of age. Quantitative research also suggests that WWID living with HIV longer delays between testing positive for HIV and registering for HIV services compared to women who report sexual transmission of HIV infection [12]. The ongoing military conflict is a risk factor that contributes to the rise in HIV with its impact influenced by numerous causal variables [13]. Injection drug use is one such factor that intensifies the conflict’s impact on the worsening epidemic situation of HIV infection [13].

Millions of patients in Ukraine require treatment for chronic diseases [14]. The bombing of healthcare facilities and pharmaceutical factories has disrupted the system for delivering and distributing medicines [3, 14, 15]. These disruptions in diagnostic and treatment programs pose a significant threat to the health of PLHIV. It has the potential to lead to deteriorating health conditions, the development of drug resistance, and an increase in the cost of treatment [14, 16, 17]. The military aggression of the Russian Federation against Ukraine in 2014 has already resulted in significant challenges for women accessing HIV services, often linked to the loss or absence of documents during displacement and bureaucratic obstracles [18].

In the context of a military conflict, the availability of services related to drug use is typically limited [19]. In 2022, 164,835 PWID received HIV prevention services in Ukraine, marking a 14% decrease compared to 2021 [20]. Opioid substitution therapy (OST) sites in Ukraine mostly continue to operate, but there have been interruptions in the delivery of medications [21, 22]. Particularly concerning is the halt in the production of OST medications at factories in Kharkiv and Odesa, raising questions about the sustainability of OST programs [17].

With the full-scale invasion of the Russian Federation into Ukraine, the migration of PWID between regions has increased the risks of HIV treatment interruption and access to HIV services in general. This heightened risk undercourses the need to enhance existing interventions and introduce new ones for WWID. These interventions should focus on early HIV detection, timely initiation of treatment, and ensuring the continuity of ART, particularly in the challenging conditions of war against Ukraine.

Unfortunately, there is a dearth of data on the specifics of implementing HIV prevention and care interventions in the context of war, especially for WWID [19, 23]. The wide array of conditions and resulting the variability in necessary responses presents significant challenges for the development and evaluation of preventive programs during military conflicts [24]. Furthermore, there is virtually no available data on how the war impact the recruitment and retention of WWID in the HIV treatment cascade. To address this scientific and practical gap, we conducted a qualitative study involving WWID in different regions of Ukraine. The aim was to identify barriers and facilitators affecting WWID’s engagement in HIV testing, treatment initiation, and ART adherence during the ongoing war against Ukraine. We anticipate that the findings from this study will serve as a robust foundation for optimizing HIV prevention and treatment programs for WWID in Ukraine in the challenging conditions of a military conflict.

## Methods

Between August and September 2022, we conducted in-depth interviews with WWID to explore their attitudes towards HIV services during the ongoing war. The study employed a qualitative descriptive approach, utilizing thematic analysis. The research was conducted in four different regions of Ukraine, specifically the cities of Kryvyi Rih and Kyiv, as well as the Ivano-Frankivsk and Odesa regions. These regions were chosen to ensure diversity in terms of the security situation and the potential movement of PWID within them.

The study participants were carefully selected based on several criteria, including their city of residence at the time of the survey, their prior experience with testing, treatment, and adherence to ART both before and during the ongoing war.

The inclusion criteria for participants were as follows: they had to be at least 18 years old, residing in one of the four designated study regions, or involuntarily displaced to the these regions after the full-scale military invasion that began on February 24, 2022. Additionally, participants should have a history of injecting drug use within the last 30 days prior to the study, and they needed to provide verbal informed consent for participating in an in-depth interview with audio recording. On the other hand, respondents who were under the influence of alcohol or drug to the extent that they could not effectively respond to interview questions or those who displayed behavior during face-to-face interviews that posed a risk to themselves or the interviewer were excluded from the study.

Recruitment of respondents was carried out by employees of HIV service NGOs, who were actively engaged in providing HIV testing and treatment services. NGO representatives approached potential participants, including both clients and non-clients, either directly or indirectly through client acquaintances and social network. Individuals who expressed interest in the study were provided with information about the research, including ‘Invitation to Participate in Study’ form. This document contained a concise overview of the study and the conditions for participation. If the potential participant was interested, they were asked to provide their contact information, including a phone number. This contact information was then shared with the interviewer responsible for contacting the potential participant, conducting a screening, obtaining verbal informed consent, and ultimately conducting the in-depth interview.

### Development of the interview guide

The semi-structured interview guide was developed in alignment with our research objectives, taking into consideration the findings from a prior study on HIV testing barriers and facilitators among PWID [25]. We conducted a pilot test of the interview guide, and the results were thoroughly reviewed and discussed by a team of researchers. These findings informed the final version of the interviewer recommendations. To cater to the language preferences of participants, we prepared two versions of the guide, both containing identical content – one in Ukrainian and the other in Russian.

### Performance of the interviews

Before the start of the interview, the respondents were screened for compliance with the study eligibility criteria. Individuals who met the inclusion criteria were asked to provide verbal informed consent. The survey was conducted by four experienced and trained interviewers either face-to-face or by telephone, depending on the remoteness of respondents’ location and security needs during martial law.

### Data management

Forms for screening and collection of sociodemographic information were completed in Ukrainian using the Qualtrics closed online platform (www.qualtrics.com). During the interviews, questions were posed in either Ukrainian or Russian, depending on the respondent’s language preference. Each interview was accompanied by digital audio recording, and these recordings were transcribed verbatim. Both the paper forms and the transcripts of the semi-structured interviews, along with the study database will be securely stored for a period of five years. After this storage period elapses, printed materials will be disposed of using a method which renders them unreadable, such as shredding. Electronic data will be securely deleted in accordance with established policies for cleansing electronic media.

### Thematic analysis

Thematic analysis was conducted in the following sequence: (1) team of three researchers discussed the content of two pilot interviews and identified the main categories for the coding matrix; (2) one researcher developed a coding matrix in Excel; (3) the team of three researchers engaged in further discussion, referring the formulation of the main categories; (4) two researchers independently pilot-coded four interview transcripts; (5) the coding results were reviewed and discussed by the entire research team, leading to final adjustments to the coding matrix.

As a result, the following coding categories were identified: (a) the impact of war on well-being and living conditions; (b) changes in drug use during wartime; (c) facilitators of engaging WWID in HIV testing, initiation of treatment, and retention on ART; (d) barriers to seeking HIV testing services, treatment initiation and retention on ART.

The subsequent coding of all transcripts was carried out in parallel by two researchers. During the coding process, they discussed the need for creating additional themes and refined their formulation. Upon completion, the two matrices were merged into a single final matrix. Throughout the analytical process, we adhered to the principle of flexibility [26] and aimed to identify both common and distinct characteristics within individual subgroups in line with the study’s objectives.

### Ethical issues

The research protocol and toolkit were approved by the Institutional Review Board No. 1 (IRB) of the charity organization “Ukrainian Institute on Public Health Policy” (official IRB registration – IRB#00007612, FWA #00029648). All respondents passed the procedure of obtaining verbal informed consent for an in-depth interview.

## Results

During the study, interviews were conducted with 38 WWID aged from 18 to 53 years old (with a median and mean age of 35 years). The group was stratified into four regions of the study: Kryvyi Rih city, Kyiv city, Ivano-Frankivsk, and Odesa regions.

### The impact of War on well-being and living conditions

The majority of respondents (34) permanently lived in the research regions. Four respondents were forced to change their place of residence due to the loss of housing or the danger of martial law. Only 10 women lived alone; the remaining 28 shared housing with their parents, other close people, and/or male partners.

### Deterioration of financial situation

All respondents noted changes in their usual lifestyle and well-being levels after the onset of the war. In January 2022, just before the war, the average personal income was 5,008 Ukrainian hryvnias (US$139), but by July 2022, it had decreased to 2,884 Ukrainian hryvnias per month (US$80). Most of them had lost their jobs even before the war, primarily due to drug use and health problems. Some had been financially dependent on their male partners for a long time. However, most of their husbands also lost their regular income due to the closure of enterprises in the winter and spring of 2022.

Women living in families with retired parents had a better financial situation, as the pension payments continued during the war. A significant proportion of the respondents received small social benefits, such as low-income or IDPs support, due to their own disabled status or a child’s disability. They also received irregular humanitarian aid from local authorities or volunteers. Temporary part-time jobs, such as seasonal vegetable sales, house cleaning, home sewing, and hairdressing services, served as additional sources of income. For several respondents, the only source of income was providing sexual services or participating in the drug trade.

### Complication of life situation

In general, during the full-scale invasion, the living conditions of all interviewees became significantly more challenging due to a decrease in income, inflation (increased prices of transportation products), the risk of shelling and stress, increased drug costs, and a deterioration of living conditions. However, the significance of individual well-being factors varied in each case. Of particular importance to WWID during the war was the quality of relationships with family members, partners, and other loved ones. If the women had loved ones, then their connections support helped them endure the hardships of wartime.

*A rocket hit our house, but from the other side, luckily. The windows were blown out. We didn’t replace them because we don’t have the money. We’re on ‘street methadone’, and we can barely afford to eat. I live with my boyfriend. He hardly walks. Lately, he understands me.* – *Kyiv, 36.*

*I have only one grandma left. I have friends, but when you have nothing, when your pockets are empty, then you’re not needed by anyone. My dear grandma helps me now, because things have become very tough. So, I’m very dependent on her now*. – *Odesa, 28.*

### Changes in drug use during wartime

The vast majority of respondents (35) had started injecting drugs before the full-scale war. Interviewees reported that changes in drug consumption mainly occurred in the first weeks of the war. An increase in the dose or frequency of drug use was primarily driven by heightened levels of anxiety, while a decrease was attributed to the difficulty of accessing drugs due to police and territorial defense control, as well as increased substances cost.

*It used to be easier before. But now, even couriers are afraid to create drug stashes. It’s hard to transport them and hard to acquire – there are checkpoints everywhere. They punish severely for it. I don’t see a way out of all this. The prices have gone up. When the war started, it was around two thousand [hryvnias]. Before the war, it was six or seven hundred.* – *Odesa, 30.*

Respondents noted the overwhelming extent of negative impact of drugs on their lives, namely on their physical and psychological state, relationships with significant people, and life prospects. However, among them there are individual participants who emphasize the positive consequences of drug use due to the reduction of stress, the opportunity to escape from uncertainty and fear during war.

*War is a shock. War is fear. War is tragedy. And to survive this, to somehow come to your senses, I guess drugs are salvation, because when I use drugs, I forget about what’s happening in life right now.* – *Kyiv, 29.*

### Facilitators of engaging WWID in HIV testing, initiation of treatment, and retention on ART

#### Social support

The presence of close relationships, support from relatives, encouragement from friends, or examples of loved ones, as well as support and accompaniment from social workers, were facilitators of accessing the entire cascade of HIV services. For a significant proportion of women, the first HIV testing took place alongside with friends, on the recommendation of volunteers and social workers at HIV/AIDS prevention sites, or during a medical examination (related to a specific somatic disease, a gynecological examination, or in places of imprisonment). If women had permanent connections with relatives, the facilitators for the initial test were often the example and encouragement of relatives (sisters, brothers) who lived with HIV. An important incentive for women to get tested for the first time was the recommendation of their male partners, who were HIV-positive or seriously ill.

*The first person who conveyed this message to me was my husband as he neared the end of his life: ‘Get tested, unless it’s too late’. – Kyiv, 40.*

Comparing the facilitators of starting ART in subgroups of women who learned about HIV infection before the war and did not start or started taking ART during the war, we found that the respondents of the second subgroup had stronger support from their loved ones and (or) motivation to care for their loved ones (child, parents, roommates). The support of trusted social workers was a significant motivator for them to start ART.

*I discussed my need to get treatment with my husband. If I do not take ART, no one will be there to bring up my child. – Odesa, 28.*

Relationships with the immediate environment, especially with partners, also facilitated the retention of WWID in therapy and their adherence to it. Taking care of loved ones and, at the same time, relying on their support in the challenging situation of war, women gained more motivation for treatment. In some cases, the breakdown of relationships led to a decrease in vitality and a devaluation of the need to take care for one’s life and continue treatment. The fear of discovering one’s HIV status due to taking ART may have also contributed to treatment discontinuation.

*Taking ART is not for fear of oneself. My niece, my mom, they ask, ‘Please take it, what will we do without you?’. – Kryvyi Rih, 41.*

*One should never cease taking ART. However, I had a dispute with my husband, so I don’t care now. If I do not return home, I will not resume therapy. I don’t want anything at all now because I used to live for the sake of my family, and now I have no family. – Odesa, 35.*

Support from social workers in HIV service NGOs was especially relevant for women who had lost family ties and were living in the worst conditions with limited material wealth. Participants of OST programs had more frequent contacts with social workers. Participation in OST programs was generally associated with increased awareness of HIV and contributed to the formation of intentions to start treatment.

*I want to be on this program [OST] now. They called me from this organization. I feel like I have support. And if it helps me, then I will definitely go and get treatment. – Kryvyi Rih, 32.*

However, as a whole, the attitudes of WWID toward OST were not unanimous. Approximately half of the respondents who received services at OST sites were committed to treatment and observed positive changes in their lives. They also reported the accessibility of HIV services. IDPs committed to OST enrolled in the program in their new place of residence, where they had more opportunities to access HIV services.

#### State of health and the threat of its loss

The risk of HIV infection and the timely initiation of HIV treatment were not equally relevant facilitators for receiving HIV services among different WWID subgroups. Other priorities took precedence in the lives of many during the war. Most of those who had not been tested for HIV previously or had a negative HIV status did not plan to be tested or were ambivalent about testing. As their drug use experience increased, some respondents became more aware of the risks associated with their lifestyle. Some mentioned they would consider testing only in the event of an immediate risk of HIV infection, such as unprotected sex or the risk of parenteral infection.

*I didn’t think it was necessary to get tested before. I thought I was healthy, in general. Although now I believe that it’s necessary to get tested every year, well, in my case. – Ivano-Frankivsk, 33.*

*If you accidentally get pricked with a needle – it often happens to us, sometimes they throw them in the mailboxes. There are syringes without caps there. That’s the kind of situation when you could really catch something. – Kyiv, 26.*

Poor health or the determination of viral load facilitated the initiation of therapy. However, as a result of improvements in immune system indicators and overall well-being, coupled with the influence of additional demotivating factors, some individuals discontinued treatment.

*I saw that my test results were bad, and it probably contributed to starting ART. Then I noticed that my cell count hasn’t gone up or down in a couple of years, and I thought if I stop taking it, nothing bad will happen. I just didn’t want to keep ruining my body. Also, with drug use, there wasn’t any time; it just got too busy and all. – Kyiv, 41.*

The interviews with WWID did not reveal key and universal individual facilitators for HIV detection, ART initiation, and retention in care during the war. In each case, a variety of circumstances and specific personal factors facilitated HIV testing, enrollment, and retention in HIV treatment. The most consistent personal facilitators for HIV services and commitment to ART were demonstrated by women who possessed a combination of several factors, including strong connections with relatives, responsibility for loved ones, family support, willingness to collaborate with specialists, a higher level of education, and a relatively prosperous financial situation. Among the social and organizational facilitators we studied, during wartime, the support and assistance of social workers from HIV services NGOs proved to be the most significant for WWID.

### Barriers to seeking HIV testing services, treatment initiation and retention on ART

#### Fear of HIV and social restrictions

Among the barriers to HIV testing, WWID most frequently cited the fear of learning their positive status, as it marks the beginning of living in constant anxiety about the future and facing the genuine threat of death. They believed that a positive HIV status would result in restrictions on sexual contacts, the disruption of relationships with loved ones, stigmatization, and a loss of life prospects. Respondents often avoided testing out of fear of a breach of confidentiality during the process and refrained from seeking treatment due to concerns that relatives might cease all communication upon learning about their illness. Given the heightened importance of permanent family and friend bonds during the war, the risk of losing them due to the revelation of a positive HIV status became an especially pertinent concern for some of the WWID.

*This [positive HIV status] hits your self-esteem hard and you get demotivated. You don’t have that energy anymore. Everything seems futile in life. – Kyiv, 32.*

*If I tell everyone that I’m going to receive pills, that I have HIV, those few of my acquaintances who still have contacts with me will scatter. The girl in whose apartment I live will kick me out. – Kryvyi Rih, 32.*

#### Drug use

This barrier, characteristic of both wartime and peacetime, becomes even more significant during times of danger such as war. Respondents noted that since the onset of the war, people have been turning to drugs more frequently as a way to cope with anxiety, causing them to care less about their own health and forego HIV testing. Many reports indicated that the war shifted priorities, making drugs more necessary but less accessible. Consequently, individuals focused primarily on finding drugs and often lacked the time to adhere to ART regimens regularly.

*I think during the war, people started using more [drugs], and they do not care about their health or whether they have HIV or not. – Kyiv, 18.*

*It just slips your mind. You set different priorities. My way of life was such that I did not care if I take [ARТ] or not. Because it’s war, and I have a headache about where to get drugs, and here, I had to go for therapy too. – Kyiv, 41.*

#### War-related stress

Among the barriers to initiating treatment, respondents also pointed to the onset of the war. However, in only a few cases could they specify the actual consequences of the war that hindered their ability to seek treatment, such as a lack of transport or money for travel. More frequently, women referred to circumstances they had heard from others *(“they say the medical center was bombed”)* or exaggerated the city’s difficult situation *(“everyone is in bomb shelters, so they can’t access their medication”)*. Some respondents perceived the war as a threat, making it seem futile to prioritize their own health in the face of the broader crisis.

*When the war started, I felt helpless, and I didn’t join the program, I didn’t start therapy. A person won’t even be able to safely go outside to get pills. – Kyiv, 36.*

Nearly all respondents who had started ART before the war and discontinued it after February 24, 2022, cited the war as the primary reason for their treatment interruption. ART discontinuation mainly occurred in the early days of the war. Due to psychological stress or rumors about difficulties in obtaining medication, they did not renew their drug supply and subsequently did not seek healthcare facilities. Two respondents discontinued ART after changing their place of residence. In reality, this discontinuation was not due to limited access to the therapy but rather because of changes in their personal lives, such as deteriorating family relationships leading to apathy and indifference towards their health or the intention to conceal their HIV status from a new partner with whom they started living after relocation).

#### Distrust of medical professionals

WWID who learned about their positive HIV status before the war but did not initiate ART reported various circumstances that hindered them from starting treatment. If they learned of their HIV infection by chance during specific medical examinations (for the treatment of chronic diseases or preparation for the termination of a pregnancy), they did not always receive proper pre-test and post-test counseling. While they were advised to undergo retesting and begin ART, their distrust in the testing results or their shock upon learning their HIV positive status often deterred them from seeking further assistance from service organizations. Under martial law, the initial mistrust of medical workers increased even more. Respondents believed that their issues were of secondary importance to doctors, who were overwhelmed due to the large number of wounded patients and were unlikely to prioritize individuals with drug addiction. Women who had lost contact with their relatives felt entirely helpless in cases of deteriorating health or illness during the war. They were certain that medical care was not available to them and, as a result, saw no point in taking care of themselves.

*During the war, it’s very difficult; there’s no time for ‘drug addicts’ and their problems now. I think any doctor would say I chose this path myself. There are a lot of wounded people now, and they have something to deal with. – Kyiv, 35.*

*Here’s the war, even if I feel really bad, I won’t call an ambulance. They won’t take me anyway. I’m not needed by anyone, so why bother. – Kryvyi Rih, 32.*

Mistrust of health workers was also a barrier to consistent ART use. WWID most frequently mentioned treatment side effects (such as sleep disturbances, nausea, diarrhea, and dizziness) as hindrances to regular ART intake and adherence to treatment. Usually, treatment discontinuation occurred when WWID did not seek advice from doctors or did not follow their recommendations.

#### Quality of communication with medical professionals

The majority of WWID had a positively assessment of the quality of work and the availability of HIV prevention services. Women who had not previously been tested for HIV reported a lack of information about the health effects of HIV and where to get tested. However, one respondent emphasized that PWID are often disinterested in receiving this information and may neglect it. Additionally, during testing, they are frequently in a state of intoxication or abstinence, and the news of infection can be shocking, making them less able to fully comprehend the information received.

Complex barriers to accessing HIV services included medical personnel’s insufficient attention to the psychological state of women during testing, a lack of persistence in conveying information about the importance of testing and initiating ART upon receiving a positive HIV status, and a deficiency in sensitivity among medical workers regarding the individual needs of WWID.

*Probably, there are enough brochures, leaflets, and booklets. But who has read them? I did not pay attention to it; I was never interested. – Kyiv, 53.*

*I was in a shock. I remember virtually nothing of what he was saying; I could not believe it can happen. – Kyiv, 29.*

Some respondents erroneously believed that the testing procedure and subsequent examinations preceding ART were paid, although this is not true. In wartime conditions, aimed a significant deterioration in their financial situation, this misconception made it impossible for them to access services. In reality, the barrier to testing in this case was a gap in informing WWID and a lack of messages tailored to the characteristics of the target audience.

*In order to get tested and find out, you have to pay 350 hryvnias. It’s tough for people now, you understand? And to find 350 hryvnias, you have to think about it, whether to buy food with them or go check your health. – Odesa, 28.*

#### Violation of the operating hours of medical institutions

WWID, particularly those residing in remote rural areas, frequently encountered challenges related to registration and other bureaucratic procedures before commencing ART. These challenges became more pronounced during the war.

*To start this therapy, one needs to get enrolled first. The red tape obstruction makes this process extremely slow. – Ivano-Frankivsk, 34.*

At the onset of the war, changes occurred in work schedules, and there were alternations in medical staff teams at certain ART sites. These challenges resulted in overcrowding of patients who had wait for extended periods to see doctors and obtain their prescribed medications. Some respondents found it burdensome to make regular visits to health facilities and maintain contact with doctors, particularly air raid alarms and when public transportation was disrupted.

*The queues for this therapy are insane. I would like to start taking ART again even now, for these services to me more accessible somehow, or even delivered by mail or in some other way. – Kyiv, 41.*

Some WWID expressed concerns about potential interruptions in the supply of ART drugs during the war. They recommended increasing the quantity of drugs dispensed and organizing delivery to patients. Respondents also highlighted the need of support from social workers during registration process and suggested improving communications between various institutions providing HIV services.

#### Residing in rural areas

The danger of shelling and disruptions in transport communications during the war significantly affected the accessibility of HIV services, as it became challenging and sometimes impossible to reach medical institutions. This barrier was especially pertinent for women living in rural areas. Simultaneously, they had less access to information about HIV and comparatively fewer facilitators for testing and initiating of treatment than urban residents. They lacked social support, as HIV service NGOs workers, even before the war, primarily operated in cities, with only occasional visits to villages. Residents of remote rural areas also expressed dissatisfaction with the limited availability of OST programs and the lack of information about these programs. WWID who had lost contact with their relatives, faced worse financial situations, and had lower motivation to care for their health were more common in rural areas. Additionally, compared to city residents, WWID living in the countryside during the war were more likely to report fears of stigmatization from fellow villagers and feelings of guilt and shame as barriers to initiating therapy and maintaining adherence.

*I didn’t make it [to the ART site]. I heard about it only once, somewhere. The workers from the NGO haven’t registered us. Can you imagine what would happen if they found out? It’s a small village. – Odesa, 40.*

## Discussion

This study provides novel insights into the impact of the war on the recruitment and retention of WWID in the HIV treatment cascade in Ukraine. Respondents diagnosed with a positive HIV status noted that the war presented a barrier to initiating ART. Virtually all respondents who discontinued ART during the war identified the war as the primary reason for discontinuation. On an individual level, the war had an indirect negative impact on nearly all stages of the HIV treatment cascade. The influence stemmed from the respondents’ perception of the situation, shifts in life priorities, and changes in readiness for individual actions during the war. Psychological tension increased, financial situation deteriorated due to job loss and other social troubles, and financial difficulties affected both WWID and their close circles of dependence. Contact with loved ones was often lost, depriving WWID of their support. The desire to seek refuge from the threats of war took precedence over taking care of their own health. WWID often relocated to safer areas, sometimes despite potential limitations on access to HIV services.

These factors’ impact is exacerbated by several other pre-existing barriers to engagement and progression through the treatment cascade, such as receiving incorrect information about the benefits of ART and other HIV services, experiencing side effects from ART drugs, facing challenges in adhering to the treatment regimen, concerns about life prospects, feeling generally well, and fear of receiving a positive HIV status diagnosis, among others.

At the organizational level, the effects of war constrained access to therapy, especially in rural areas. Work schedules of healthcare facilities constantly change, medical personnel face redundancy, transportation is disrupted, and opportunities to regularly and promptly access healthcare facilities are limited. Air raid alarms can halt service provision, and interruptions in the supply of ART drugs are possible, leading to queues when seeking assistance. Concurrently, pre-existing organizational barriers unrelated to the war persist, including the potential for privacy violations, stigma, and a lack of up-to-date information about the benefits of HIV testing and services contingent on the diagnostic result.

It is challenging to isolate any major barriers to engagement in HIV testing, treatment initiation, and retention in services. However, there are several barrier components that have emerged as a result of war, or whose impact has often intensified in the context of danger and social hardship, where other basic needs typically take precedence over the need for HIV testing and treatment. In times of conflict, priority needs include food, shelter, water, and security [24]. Military and humanitarian needs brought about by war diminish the priority of HIV services,[17, 21] while, conversely, the importance of primary care rises [24].

Based on this, it can be assumed that during the war, the facilitators of engagement in HIV services become even more critical than in the pre-war period. This means that the additional challenges linked to the repercussions of military aggression should take precedence. Strong connections with relatives, family and friends’ support, a relatively stable financial situation, a commitment to ART, poor health, social responsibility, and a high level of trust in ART all facilitate the use of services and retention in them. Among these factors, the significance of family bonds, support from social workers, and poor health (feeling unwell) were most frequently emphasized.

Respondents’ attitudes towards OST varied widely, from satisfaction with the use of these services and an awareness of the positive impact of OST on adherence to ART and improvements in their own lives, to low awareness of OST programs, complaints from residents of remote rural areas about the inaccessibility of OST and the lack of information about these programs. Some respondents also viewed OST drugs as harmful to health. During times of war, OST programs endeavor to address the specific needs of their patients [21].

Military conflicts are dynamic emergency situations, which makes determining appropriate response procedures challenging. These procedures must align with the evolving context, particularly considering the stage of the military conflict’s development [24]. Our proposals are formulated in the context of the active stage of the war against Ukraine and pertain to optimizing prevention and treatment programs for WWID amidst ongoing migration processes, the destruction of infrastructure, deteriorating access to social conditions, and medical services.

Considering the active movement of people, it is crucial to ensure the extension of service access in a new territory and facilities. Respondents highlighted the importance of dispensing ART drugs for longer durations, which is especially significant given transportation challenges and the unstable working conditions of healthcare facilities. With the emergence of new clusters of HIV infection, preventive measures are becoming exceedingly relevant among IDPs [27]. One distinctive feature of the current situation is the unpredictability of its duration, necessitating the development of long-term interventions, territorial decentralization, and the integration of HIV prevention and treatment services, including harm reduction services, particularly OST [17]. Research has shown that integrating HIV services with services for drug users does not increase stigma associated with a positive HIV status or drug use; instead, it is perceived by service recipients as offering higher quality [28]. Moreover, patients at integrated care sites more frequently reported receiving ART and referrals to additional services [28]. In general, the integration of services related to drug use with other medical services, such as primary care and psychological support, can reduce stigma levels, eliminate existing access barriers, and broaden the range of available services [19]. During times of the war, the need for intervention in the field of mental health and psychosocial support [2] becomes urgent, especially among IDPs and women [29]. Additionally, the role of social support increases, particularly for IDPs who lack complete and up-to-date information about services access in their new environment. According to reviews, the provision of supervised services improves the health and well-being of PWID [30].

## Conclusions

The war in Ukraine persists and continues to exacerbate its negative impact on all aspects of the population’s lives, especially WWID. Organizers and providers of HIV services must exert every effort to sustain and optimize these services, considering the evolving context and requirements. The shifting life situations and changing priorities of WWID underscore the necessity for a dynamic and comprehensive assessment of existing challenges.

### Limitations

First, it’s important to note that the respondents in this study were selected using convenient method. Only four respondents reported forced relocation, which means that the results may not be fully representative of the entire community of WWID. Further studies should place a stronger focus on internally displaced WWID population. However, the study’s stratification by four territories and eight subgroups based on their stage in the treatment cascade, combined with the qualitative design using in-depth interviews, allowed for a fairly comprehensive understanding of the barriers and facilitators for the engagement and retention of WWID in HIV services during the war against Ukraine.

Second, the field phase of the study was conducted in a cross-sectional format over two months in 2022. During this research period, the war against Ukraine was in its active phase, and therefore, the results obtained are pertinent to the prevailing situation at that time. The inclusion of retrospective questions in the interview guide, particularly to the way of life and attitudes towards HIV services that were characteristic of the respondents before the onset of the war, allowed for a certain comparison and a separate assessment of the influence of factors directly related to the war on the treatment cascade. Ukrainians have a strong belief in and aspiration for victory, so defining relevant research tasks for the post-active and recovery stages is a promising avenue for future research.

Finally, it’s essential to note that we did not examine the attitudes of service providers, particularly social and health workers. Undoubtedly, the opinions of WWID receiving HIV services are a primary focus in such studies. However, in order to obtain a more comprehensive understanding, especially regarding the influence of organizational factors, it is imperative to explore the perspectives of service providers in subsequent stages of research.
